# The relationship between adverse childhood experience, resilience, and psychosocial well-being among undergraduates in Osun State, Nigeria

**DOI:** 10.1186/s40359-026-04530-5

**Published:** 2026-04-14

**Authors:** Anuoluwapo Precious Opadele, Bisola Odunayo Alabi, Oyindamola Bolorunduro, Bukola Emilola Olorunfemi, Oyindamola Ayoola, Temidayo Akinreni, Oladayo Akinwale Damilola, Olayinka Ajao, Ifedola Olabisi Faramade, Akinlolu Omisore

**Affiliations:** 1 Graduates of BSc. Public Health program, Osun State University, Osogbo, Nigeria; 2 Faculty of Nursing Sciences, Osun State University, Osogbo, Osogbo Nigeria; 3 Department of Community Medicine, Osun State University, Osogbo, Nigeria

**Keywords:** ACE, Childhood, Psychosocial, Resilience, Well-being, Mental health

## Abstract

**Background:**

Adverse childhood experiences (ACEs) are potentially traumatic events that are associated with negative outcomes, which may contribute to a life course trajectory that connects early adversity with long-term adult health issues. Our study assessed the relationship between ACE, resilience, and psychosocial well-being among undergraduates in tertiary institutions in Osun State, Nigeria.

**Methods:**

This study utilized a descriptive cross-sectional research design. The sample size was calculated using the Leslie Kish Formula, and the sampled population consisted of 514 students from two selected universities in Osun State. A multistage sampling method was used for the study, and a modified questionnaire adapted from the WHO ACE IQ tools was used for data collection. Data collected were analyzed using descriptive statistics of frequency, percentage, means, and standard deviation for univariate analysis, while inferential statistics of chi-square were used for bivariate analysis.

**Results:**

More than half (56.2%) of the respondents had experienced moderate to severe ACE, while 44% had mild or no ACE. Two-thirds of the respondents (66%) had an exceptional level of resilience. There was a statistically significant association between the level of resilience and the experience of ACE (*P* = 0.028; χ2 = 7.174; Confidence Interval = 1.170, 25.880), as more respondents who had no/mild ACE exhibited an exceptional level of resilience. Similarly, the psychosocial well-being was statistically significant with the level of resilience among all respondents (*P* < 0.001; χ2 = 19.737; Confidence Interval = 1.840, 41.610).

**Conclusion:**

While ACE is prevalent among undergraduates in Osun state, the level of resilience and psychosocial well-being was largely exceptional and good/excellent, respectively. Our findings suggest that ACEs are common among university students in Osun State, Nigeria. This highlights the urgent need for targeted prevention strategies that foster resilience and improve psychosocial well-being among this population.

**Supplementary Information:**

The online version contains supplementary material available at 10.1186/s40359-026-04530-5.

## Background

Adverse childhood experiences (ACEs) are traumatic events that occur in childhood, which can have negative effects on health in later years [1–4]. Felitti et al., (1998) [5] categorized these events into ten groups, which include child abuse (physical, emotional, and sexual abuse), neglect (physical and emotional neglect), household dysfunction/divorce, incarcerated relative, substance abuse, mental illness, and violence [6–8]. According to Manyema and Richer [9] in 2019, exposure to ACEs arises from a multifaceted interaction of various risk and protective factors, which stem from individual, social, community, and societal levels [9]. Previous research indicates that physical maltreatment, emotional neglect, and challenges stemming from household instability are the most frequently reported types of ACEs [4, 10–12].

Previous studies revealed varying prevalence rates of Adverse Childhood Experiences (ACEs) across different age groups and regions. Among children aged 0-7years, 22.6% have experienced two or more of the ten categories of ACEs, while 20.3% of adolescents have been exposed to four or more ACEs [1, 2, 13]. Additionally, ACEs are associated with negative outcomes such as poorer mental health during childhood, reduced educational attainment, and increased anti-social or violent behaviours [14–16]. These factors may contribute to a life course trajectory that connects early adversity with long-term adult health issues [6]. In Nigeria, a study by Oladeji et al. [17] in 2010 found a prevalence rate of 46.2% among adults [17], while rural populations in Uganda and Tanzania reported ACE exposure rates as high as 90% [11, 18]. According to another study, approximately one-third (67.8%) of students have experienced ACEs [19]. In Nigeria, physical neglect is identified as the most prevalent form of ACE, with high rates observed among young people in low-income urban areas and university communities [20, 21]. ACEs among university students are linked to various mental health issues, which can negatively impact academic performance [22–24]. This can lead to diminished academic achievement, which not only impacts the individual’s productivity but also has broader economic consequences for the nation [13].

The ACEs framework by Felitti et al., (1998) [5], described the correlational relationship between ACEs and health outcomes. A key component of the framework is the cumulative “ACE score,” which is related to an increased likelihood of a range of resilience and psychosocial well-being. In spite of the known effects of ACEs, some individuals overcome challenging circumstances, leading to the concept of resilience [25, 26]. Within this correlational framework, resilience is conceptualized not as a mediating mechanism, but as a parallel, associated construct that coexists with ACE exposure and related outcomes. While ACEs capture cumulative exposure to adversity, resilience reflects the capacity for positive adaptation despite such adversity. Importantly, resilience does not negate the effects of ACEs nor function here as an explanatory pathway; rather, it is examined as a co-occurring attribute that may vary independently or in relation to ACE scores. Resilience refers to the ability to adapt positively in the face of adversity, trauma, or significant stressors, such as family issues, health challenges, or financial strain [13, 27, 28]. Although the propensity for resilience may be genetically and neurobiologically related, it can also be influenced by environmental factors [29, 30]. Psychosocial well-being encompasses the development of cognitive, emotional, and spiritual strengths within individuals, families, and communities, which fosters overall positive social relationships [31].

Although the concepts of ACEs and resilience in relation to psychosocial well-being are better understood, measuring them remains complex and challenging. The relationship between these three factors, adverse childhood experiences, resilience, and psychosocial well-being, has not been thoroughly explored, particularly among Nigerian youths. In this study, the ACEs framework provides a theoretical foundation for this gap by defining and examining the correlational connections between ACEs, resilience, and psychosocial well-being among undergraduates in tertiary institutions in Osun State.

## Method

### Study design and population

This cross-sectional correlational study was carried out in two tertiary institutions in Osun State, Nigeria- Osun State University and Adeleke University. Osun State was purposively selected due to its blend and abundance of public and private universities, which provided a representation of diverse student populations. Also, despite the existing availability of prevalence data in neighbouring states [32, 33], there is a dearth of data on the prevalence of ACEs in Osun State among university students. The limited data focused on a school survey of children aged 6–16 years [34]. The target population consisted of undergraduates aged 18 years and above, enrolled in various departments across the selected universities.

### Sample size and sampling procedure

The sample size was determined using the Leslie Kish formula with an ACE prevalence of 54% [35]; q = 1-p; e = 0.05. The calculated sample size was 382. To account for potential non-response and to facilitate cross-tabulation in the analysis, an additional 35% was added, resulting in an approximate sample size of 516. Therefore, 128 and 388 undergraduates were recruited from Adeleke University and Osun State University, respectively.

Participants were selected through a multistage sampling technique that involved first the selection of universities amongst the ten universities in Osun state using a simple random sampling method via balloting. This led to the selection of Osun State University and Adeleke University. Faculties and departments in the selected universities deemed eligible based on student population size and relevance to the study objectives were listed, and a simple random sampling via balloting was used to select participating faculties. In the third stage, departments within the selected faculties formed the sampling frame. Departments were similarly selected using a simple random sampling via balloting. In the final stage, undergraduates were recruited from selected departments across the two institutions using a simple random sampling method via balloting after a proportionate allocation of the number of students to be taken from each institution was done.

## Data collection tools and procedure

### WHO’s ACE International Questionnaire (ACE-IQ)

We leveraged the widely used WHO’s ACE International Questionnaire (ACE-IQ) to assess the experience of ACEs [36]. The instrument contains 43 items designed to measure an individual’s experience and severity of ACEs [36]. A validation study by Kazeem (2015) [37] in Nigeria, a Cronbach’s α of 0.80 was reported for all items of the ACE IQ, demonstrating acceptable reliability in this context [37]. Furthermore, this instrument has been widely validated and employed in different contexts in countries, including sub-Saharan Africa and Nigeria, which ensured its application within local contexts [37–39]. The instrument was adapted and divided into five sections; Section A comprised 12 items on socio-demographic characteristics and relationship dynamics; Section B consisted of 13 items on ACEs with four Likert scales ranging from ‘never (0)’ to ‘regularly’ (5) in ascending order.

### Resilience scale

The Resilience Scale was developed by Wagnild and Young (1993) [40], comprising a 17-item rated on a 7-point Likert scale, across five domains: characteristics of meaningful life (purpose), perseverance, self-reliance, equanimity, and existential aloneness [40]. The instrument provides a composite score of resilience, with higher scores indicating greater resilience [40]. Its validity and reliability have been demonstrated in several contexts, including among university students in Nigeria, where Abiola and Udofia (2011) reported a Cronbach’s alpha coefficient of 0.87 [41], and in Japan, where Nishi et al. (2010) likewise established its reliability [42].

### Psychosocial well-being scale

The Ryff’s 52-item psychosocial well-being scale was adapted for this study [43]. The instrument’s reliability and validity have been confirmed in diverse settings, including Iran, where Khanjani et al. (2014) reported satisfactory psychometric properties [44] and Nigeria, where Igbokwe et al. (2016) reported a Cronbach’s alpha of 0.84 [45].

The adapted instruments were pretested using 10% of the sample size in another university with similar characteristics, but not part of the study settings. The pretested instruments were administered to respondents in their classrooms during class time, lasting about 20–25 min, under the supervision of the researchers and two research assistants. A copy of the final instrument is attached to Appendix 1.

### Data analysis

The data were analyzed using an association-based analytical framework consistent with the exploratory aim of examining the relationship among adverse childhood experiences (ACEs), resilience, and psychosocial well-being among undergraduates via SPSS 23.0, and appropriate univariate and bivariate analyses were conducted. At the univariate level, descriptive statistics of the frequencies table and charts, mean & standard deviation were used to present categorical and continuous variables, respectively. At the bivariate level, chi-square tests were used to assess the association between adverse childhood experiences, resilience, and current psychosocial well-being. Multivariable modeling was not conducted because the primary objective of the study was to identify and describe associations between key constructs rather than to estimate adjusted effects or develop predictive models. Therefore, the use of bivariate analysis approach was intentional and grounded in the exploratory nature of the study, which sought to identify preliminary patterns of association between key constructs rather than to estimate independent effects or test causal hypotheses. Also, this approach was considered appropriate for addressing the study objectives.

Similar to previous studies, the score measures for ACE, resilience, and psychosocial well-being are highlighted below: Moderate or high level of adverse childhood experiences (5–13), while mild or no adverse childhood experiences (0–4) [7]. Scores of resilience include: A developing level of resilience (0–37), an established level of resilience (38–43), a strong resilience (44–48), an exceptional resilience (49–60) [46]. Scores for psychosocial well-being include: Poor or fair well-being (4–12) and good/excellent well-being (13–28). Five hundred and sixteen questionnaires were administered; only two (2) were not properly filled and not suitable for analysis, hence 514 were coded and analyzed, making a 99.6% response rate.

### Ethical considerations

Our study was conducted in line with the 2013 Declaration of Helsinki on research involving human subjects [47]. This study was approved by the Health Research Ethics Committee (HREC) at Osun State University, Osogbo, with approval number: UNIOSUNHREC2020/PBH/010. Permission to conduct the study was also obtained from the Heads of the departments at the selected universities involved in the study. Prior to data collection, the study’s objectives, significance, and benefits were explained to participants. They were assured that participation in the study was voluntary and that they could leave at any time without consequences. To ensure anonymity, all personally identifiable information were removed from the dataset, and responses were anonymized upon data completion. Additionally, participants’ written consent was obtained before data collection. The data obtained from this study were secured in an online database accessible only to the principal investigator and study researchers.

## Results

The demographic characteristics of the participants are presented in Table 1. The majority of participants were female (65.6%), with a mean age of 20 years (SD = 2.42). Most respondents (92.4%) were categorized as youth (18–24 years). Regarding religious affiliation, the majority identified as Christians (71.6%), followed by Muslims (27.4%).

**Table 1:** Socio-demographic characteristics of respondents (*n* = 514)

Variables	Frequency	Percentage (%)
Sex		
Male	177	34.4
Female	337	65.6
**Age -group in years (Mean age 20 ± 2.42 Min = 18**,** Max = 30)**		
Youth (18–24 years)	475	92.4
Young adult (25–30 years)	39	7.6
**Religion**		
Christianity	368	71.6
Islam	141	27.4
Traditional	4	8.0
None	1	2.0
**Ethnicity**		
Yoruba	448	87.2
Igbo	33	6.4
Niger-delta	17	3.3
Others	16	3.1
**Parental marital status**		
Currently married	445	86.6
Not currently married	69	13.4
**Grew up living with parents**		
Yes	474	92.2
No	40	7.8
**Type of family**		
Monogamous	416	80.9
Polygamous	98	19.1

**Fig. 1:**
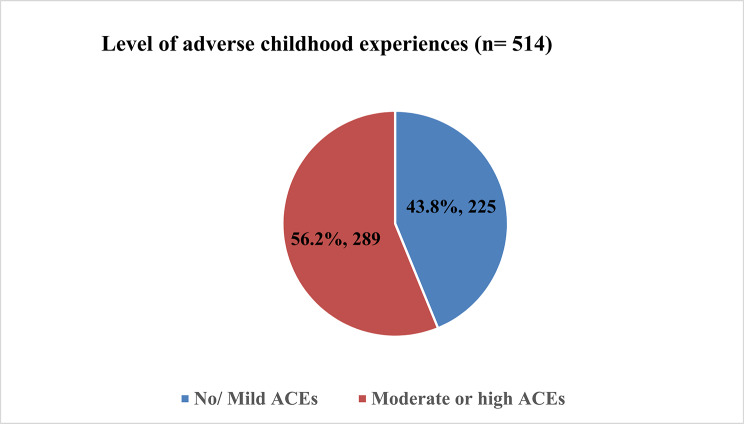
Level of adverse childhood experiences (*n* = 514)

Figure 1 shows that a higher proportion of respondents had experienced moderate or high levels of adverse childhood experiences, 289 (56.2%), compared to those who had experienced mild or no adverse childhood experiences, 225 (43.8%).

**Fig. 2:**
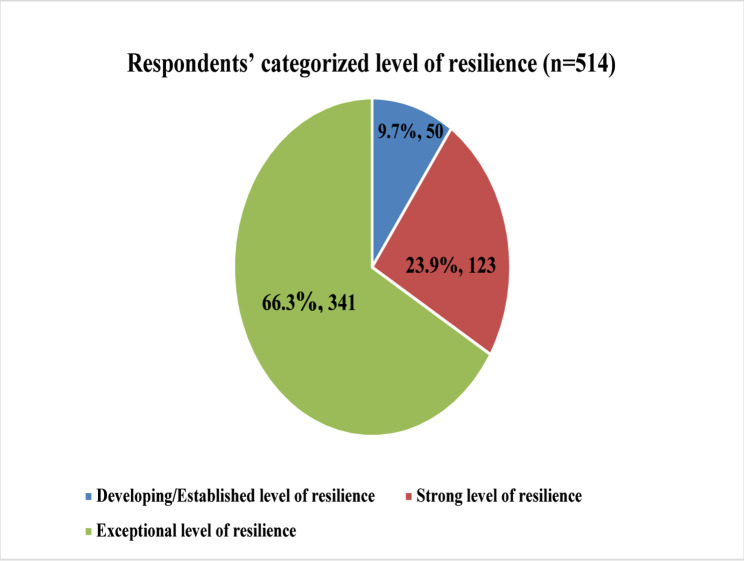
Respondents’ categorized level of resilience (*n* = 514)

Figure 2 shows the level of resilience displayed by the respondents. A large percentage, 341 (66.3%) of respondents exhibited an exceptional level of resilience. This was followed by respondents who exhibited strong resilience 123 (23.9%), and lastly, a developing/established level of resilience 50 (9.7%).

Table 2 shows the relationship between the respondents’ level of ACE and the different levels of resilience, and it shows a statistically significant relationship (*P* = 0.028). More of the respondents who had no/mild ACE exhibited an exceptional level of resilience, 157 (69.8%), and a strong level of resilience, 5.5 (24.4%), compared to those who had moderate/high ACE, 184 (63.7%), and 68 (23.5%), respectively. Likewise, more of the respondents who had moderate/high ACE exhibited developing/established resilience, 37 (12.8%), compared to those who had no/mild ACE, 13 (5.8%).

**Table 2:** Respondents’ ACE category measured against respondents’ resilience category (*n* = 514)

ACE category	Resilience Category			Statistics Indices [95% Confidence Interval (CI)]
	Developing/established Resilience	Strong Resilience	Exceptional Resilience	
No/mild ACE	13(5.8%)	5.5(24.4%)	157(69.8%)	χ2 = 7.174 *P* = 0.028* [1.170, 25.880]
Moderate/high ACE (^a^)	37(12.8%)	68(23.5%)	184(63.7%)	

* Statistically significant at *p* < 0.05, Significance level was set at 95%

(^a^) indicates the reference category for each predictor variable

Table 3 above shows the relationship between respondents’ ACE levels and their psychosocial well-being. More of those who had experienced No/mild ACE had good/excellent psychosocial well-being 150 (66.7%), than those who had moderate/high ACE 188 (63.3%). Similarly, more of those who had experienced moderate/high ACE had poor/fair psychosocial well-being 106 (36.7%) than those who had no/mild ACE 77 (33.3%). However, this relationship was not statistically significant.

**Table 3:** Respondents’ ACE category measured against respondents’ psychosocial well-being status (*n* = 514)

ACE category	Psychosocial well-being status		Statistics Indices [95% Confidence Interval (CI)]
	Poor/fair well-being	Good/excellent well-being	
No/mild ACE	77(33.3%)	150(66.7%)	χ2 = 0.620 *P* = 0.431 [0.435, 1.960]
Moderate/high ACE (^a^)	106(36.7%)	188(63.3%)	

* Statistically significant at *p* < 0.05, Significance level was set at 95%

(^a^) indicates the reference category for each predictor variable

In Table 4 above, there is a statistically significant association between the level of resilience and the level of psychosocial well-being (p < 0.001). More respondents who exhibited a developing/established level of resilience showed poor/fair psychosocial well-being 30 (60.0%), compared with 50 (40.6%) and 101 (29.6%) of those with strong and exceptional resilience, respectively. Alternatively stated, seven out of ten respondents with exceptional resilience had good/excellent psychosocial well-being, compared to six and four out of ten for those with strong and developing/established resilience.

**Table 4:** Respondents’ resilience category measured against their psychosocial well-being (*n* = 514)

Resilience category	Psychosocial well-being status		Statistics Indices [95% Confidence Interval (CI)]
	Poor/fair	Good/ excellent	
Developing/established Category	30(60.0%)	20(40.0%)	χ2 = 19.737 *P* < 0.001* [1.840, 41.610]
Strong category	50(40.6%)	73(59.4%)	
Exceptional category (^a^)	101(29.6%)	240(70.4%)	

* Statistically significant at *p* < 0.05, Significance level was set at 95%

(^a^) indicates the reference category for each predictor variable

## Discussion

Research has demonstrated that young adults experience at least one of the nine adverse childhood experiences at some point in their lives [6, 27, 48, 49]. These experiences include physical, verbal, sexual abuse, emotional neglect, abuse of alcohol or drugs, mental illness, witnessing a mother being abused, losing a parent to divorce or separation, at one stage of their life or another other [4, 6]. While many individuals manage to build substantial resilience and adapt positively despite such challenges, others struggle to recover from the long-term effects [50, 51]. The presence of ACEs, coupled with an individual’s ability or inability to develop resilience, plays a significant role in shaping their psychosocial well-being [26, 50].

Findings from our study showed that more than half of the respondents had experienced moderate to high ACE. Previous studies had also shown a nearly equivalent prevalence rate to our study. For instance, a 2020 study conducted in Ethiopia found that ACEs were notably common, with a prevalence rate of 50.7% [52]. Similarly, a large-scale study in Nigeria found that 46.2% of adults reported experiencing ACEs [17]. Another Nigerian study also showed that one-third (67.8%) of the participants had experienced one or more ACE [19]. This similarity in the experiences of ACE among adolescents, pupils, and undergraduates further emphasizes the prevalence of ACE in society. However, there are notable regional variations. Studies conducted among rural populations in Uganda and Tanzania revealed alarmingly higher prevalence rates, reaching up to 90% [11, 18]. This suggests that cultural and environmental factors may influence the reporting and experience of ACEs. The prevalence in other regions further reflects these differences. For instance, in Vietnam, a study involving university students found that over three-quarters (76.2%) had experienced ACEs [53]. Moreover, research involving Brazilian university students reported a prevalence of 74.4% [54], but approximately one-third (36.1%) among participants in Austria [55]. These findings demonstrate that ACEs are prevalent in diverse societal contexts.

Additionally, as regards the level of resilience and its relationship with ACE, a tenth of the respondents had a developing/established level of resilience, while about a quarter and two-thirds had a strong and exceptional level of resilience, respectively. Thus, at least nine out of ten respondents had strong to exceptional resilience. This may not be unexpected, as more than half of the respondents had moderate/ high ACE. These results are consistent with a study conducted in Wales, Europe, which reported a high prevalence of resilience among its participants, with 48.3% demonstrating robust resilience [56]. A similar pattern was identified in a study among youths in Taiwan, which revealed a statistically significant relationship between resilience and ACE exposure [57]. Additional studies from Turkey and the USA have also described a significant correlation between ACE scores and resilience [58, 59]. These studies further reported that an increase in ACEs correlated with a higher level of resilience [58, 59]. Moreover, a 2014 study by Bethell et al. [60] in the USA found that 48.4% of children who exhibited resilience had also experienced ACEs [60]. This finding suggests a potential relationship whereby individuals who reported early adverse experiences also tended to report higher levels of resilience [60]. While the cross-sectional nature of the study does not allow causal inferences, the observed association may indicate that exposure to adversity is linked with variations in psychosocial coping resources among individuals [3].

Regarding the relationship between ACE and psychosocial well-being among the respondents, there was no significant correlation between exposure to ACE and psychosocial well-being (*P* = 0.431). This is similar to the result of a study conducted in Nigeria, which showed no significant association between ACE and psychosocial factors [19]. However, this is in variance with a study among Chinese university students, where a significant association was found between ACE and psychosocial well-being [61]. More of those who had experienced ACE exhibited poorer psychosocial well-being compared to those who had not been exposed to such experiences [61]. However, there was a significant correlation between the level of resilience and the level of psychosocial well-being in our study, as the result shows that two-thirds of the respondents who had moderate/high ACE also exhibited an exceptional level of resilience, comparable to findings from a study in Iran, which showed a statistical relationship between resilience and psychosocial well-being [62].

One plausible implication of these results is that ACEs remain prevalent among young people, including undergraduates. The findings also indicate that a greater proportion of participants who reported experiencing ACEs demonstrated exceptional levels of resilience. This pattern suggests a correlational relationship between ACEs and resilience and highlights the potential role of resilience in relation to psychosocial well-being. However, given the cross-sectional and correlational nature of the study, these findings should be interpreted as associations rather than causal relationships. Further studies conducted in diverse settings are recommended to explore the relationships among ACEs, resilience, and psychosocial well-being.

### Strength of the study

Our study explored the correlational relationship between adverse childhood experiences, resilience, and psychosocial well-being, which have not been thoroughly explored, especially among university students in Osun State, Nigeria. Findings from our study provide valuable insights into how resilience may be related to the negative effects of ACEs among university students.

### Limitations and future directions

Our study has several limitations. First, our study’s sample was limited to students from only two universities in Osun State, which may not fully represent the broader student population. Therefore, we encourage further longitudinal research to explore how the relationship between ACEs, resilience, and psychosocial well-being evolves. Also, the study employed a cross-sectional correlational design to explore the experience of ACEs; we recognized this may contribute to reporting and recall bias.

Furthermore, the absence of multivariate analysis ensures that we were unable to account for potential confounding variables that may influence the observed relationships. Also, the grouping of continuous scale scores for ACEs, resilience, and psychosocial well-being into categorical groups for chi-square analysis, although guided by established scoring thresholds in the literature, we believe this may have reduced statistical power and limited the ability to detect more nuanced relationships among variables. Hence, we employ future studies to consider multivariable analytical approaches that retain continuous scale properties and adjust for relevant covariates.

### Implications

Our study has several implications for research and intervention. First, our findings suggest that ACEs are common among university students in Osun State, Nigeria. This highlights the urgent need for targeted prevention strategies that foster resilience and improve psychosocial well-being among this population. These findings further hold important cultural implications, especially within the Nigerian context, where the nuclear family, religious institutions, and communities shape ACEs. Therefore, universities, in collaboration with trained mental health first aiders or professionals, family, religious, and community leaders, can play a proactive role in designing preventive programs geared towards creating safe spaces while reducing stigma and trauma associated with ACEs.

Given the substantial impact of ACEs on students’ mental and emotional health [62], it is pertinent that awareness campaigns that focus on the dangers of ACEs should be implemented. These efforts should target not only students in the academic communities but also their families and the broader society. Interventions could incorporate student-led initiatives designed to enhance psychosocial well-being through peer support and educational activities. Moreover, policymakers and stakeholders in universities could develop comprehensive programs addressing the broader spectrum of challenges faced by youths, particularly those stemming from ACEs.

## Conclusion

Our study represents an effort to explore the relationship between ACEs, resilience, and psychosocial well-being among university students in Osun State, Nigeria. ACEs were prevalent in our study, which highlights the need for greater attention to this issue. Resilience was found to be statistically significant in relation to ACEs and emerged as a major factor contributing to psychosocial well-being, particularly for individuals who have experienced or are at risk of ACEs. However, there was no significant association between direct exposure to ACEs and psychosocial well-being. These findings suggest that resilience may play a protective role, mitigating the negative effects of ACEs on students’ mental and emotional health.

## Supplementary Information

Supplementary Material 1.

## Data Availability

All data relevant to the study are included in the article or uploaded as supplementary information.

## Abbreviations

ACEs: Adverse Childhood Experiences
ACE-IQ: ACE International Questionnaire
HREC: Health Research Ethics Committees
SSA: Sub-Saharan African
