# Supplementary material for: The relationship between adverse childhood experience, resilience, and psychosocial well-being among undergraduates in Osun State, Nigeria
Source: BMC Psychol. 2026 Apr 14;14:764. doi: 10.1186/s40359-026-04530-5 (PMC13188633; doi:10.1186/s40359-026-04530-5)
Supplement: Supplementary file 1 — Supplementary Material 1. [file 40359_2026_4530_MOESM1_ESM.docx]

**APPENDIX 1**

**THE RELATIONSHIP BETWEEN ADVERSE CHILDHOOD EXPERIENCES, RESILIENCE, AND PSYCHOSOCIAL WELL-BEING OF UNDERGRADUATES IN OSUN STATE**

**Dear respondent,**

We are collecting information on the above for our project. We implore you to give as much valid information as possible, and all information will be kept confidential. Thanks. Questionnaire Code …….

**SECTION A: SOCIODEMOGRAPHIC CHARACTERISTICS& RELATIONSHIP DYNAMICS**

1. Sex 1. Male ( ) 2. Female ( )
2. Age (as at last birthday) ………………
3. College…………………………………
4. Faculty ………………
5. Department ………………………
6. Religion 1. Christianity 2. Islam 3. Traditional 4 other specify ………………
7. Ethnicity 1. Yoruba 2. Igbo 3. Niger Delta 4. Other specify ……
8. Mother’s highest education attainment 1. None 2. Primary 3. Secondary 4. Tertiary
9. Father’s highest education attainment 1. None 2. Primary 3. Secondary 4. Tertiary
10. Parental marital status 1. Married 2. Divorced 3. Separated 4. Never married 5. Widowed
11. Grew up with 1. Parents 2. Relatives 3. Guardian 4. Others- …………………….
12. Type of family - 1. Monogamous 2. Polygamous

**SECTION B: ADVERSE CHILDHOOD EXPERIENCES:** These questions refer to adverse events you experienced while growing up till 18 years of age.

| **S/N** | **Item** | **Never** | **Rarely** | **Some**  **Times** | **Often** |
| --- | --- | --- | --- | --- | --- |
| 13 | Did your parent or other adult in the household often swear at you, insult you, or act in a way that made you afraid that you might be physically hurt? |  |  |  |  |
| 14 | Did your parent or other adult in the household often push, grab, or ever hit you so hard that you had marks or were injured? |  |  |  |  |
| 15 | Did an adult or person at least 5 years older than you ever touch or fondle you to have oral, anal, or vaginal sex with you? |  |  |  |  |
| 16 | Did you often feel that no one in your family loved you or supported each other? |  |  |  |  |
| 17 | Did you often feel that you didn’t have enough to eat, had no one to protect you, or your parent were too drunk or high to take care of you? |  |  |  |  |
| 18 | Does an adult or person at least 5 years older than you ever attempt to have oral, anal, or vaginal sex with you? |  |  |  |  |
| 20 | How often do your parents or caregivers ignore or fail to get you medical treatment when you are sick or hurt? |  |  |  |  |
| 21 | Do you feel that someone in your family hates you? |  |  |  |  |

| **S/N** | **Item** | **Many times** | **A few times** | **Once** | **Never** | **Refused** |
| --- | --- | --- | --- | --- | --- | --- |
| 22 | Did you see or hear a parent or household member in your home being yelled at, screamed at, sworn at, insulted or humiliated? |  |  |  |  |  |
| 23 | Did you see or hear a parent or household member in your home being slapped, kicked, punched or beaten up? |  |  |  |  |  |
| 24 | Did you see or hear a parent or household member in your home being hit or cut with an object, such as a stick (or cane), bottle, club, knife, whip etc.? |  |  |  |  |  |
| 25 | Did a parent, guardian or other household member yell, scream or swear at you, insult or humiliate you? |  |  |  |  |  |
| 26 | Did a parent, guardian or other household member threaten to, or actually, abandon you or throw you out of the house? |  |  |  |  |  |
| 27 | Did a parent, guardian or other household member spank, slap, kick, punch or beat you up? |  |  |  |  |  |
| 28 | Did a parent, guardian or other household member hit or cut you with an object, such as a stick (or cane), bottle, club, knife, whip etc? |  |  |  |  |  |
| 29 | Did someone touch or fondle you in a sexual way when you did not want them to? |  |  |  |  |  |
| 30 | Did someone make you touch their body in a sexual way when you did not want them to? |  |  |  |  |  |

| **S/N** | **Items** | **Yes** | **No** |
| --- | --- | --- | --- |
| 31 | Did you live with a household member who was a problem drinker or alcoholic, or misused street or prescription drugs? |  |  |
| 32 | Did you live with a household member who was depressed, mentally ill or suicidal? |  |  |
| 33 | Did you live with a household member who was ever sent to jail or prison? |  |  |
| 34 | Were your parents ever separated or divorced? |  |  |
| 35 | Did your mother, father or guardian die? |  |  |

**SECTION C: RESILIENCE-**

| **S/N** | **Items** | **Strongly**  **Agree** | **Agree** | **Un-decided** | **Disagree** | **Strongly**  **Disagree** |
| --- | --- | --- | --- | --- | --- | --- |
| 36 | I feel proud that I have accomplished things in life. |  |  |  |  |  |
| 37 | I usually take things in stride (step by step) |  |  |  |  |  |
| 38 | I am friends with myself (love myself) |  |  |  |  |  |
| 39 | I feel that I can handle many things at a time. |  |  |  |  |  |
| 40 | I can get through difficult times because I've experienced difficulty before |  |  |  |  |  |
| 41 | I have self-discipline |  |  |  |  |  |
| 42 | I can usually find something to laugh about |  |  |  |  |  |
| 43 | My belief in myself gets me through hard times. |  |  |  |  |  |
| 44 | In an emergency, people can generally rely on me. |  |  |  |  |  |
| 45 | When I'm in a difficult situation, I can usually find my way out of it. |  |  |  |  |  |
| 46 | I usually manage/cope one way or another. |  |  |  |  |  |
| 47 | I am interested in things (follow things through). |  |  |  |  |  |

**SECTION D: PSYCHOSOCIAL BEHAVIOUR**

| **S/N** | **Items** | **Strongly agree** | **Agree** | **Strongly disagree** | **Disagree** |
| --- | --- | --- | --- | --- | --- |
| 48 | I like most parts of my personality.” |  |  |  |  |
| 49 | “When I look at the story of my life, I am pleased with how things have turned out so far.” |  |  |  |  |
| 50 | “The demands of everyday life often get me down. |  |  |  |  |
| 51 | “In many ways, I feel disappointed about my achievements in life. |  |  |  |  |
| 52 | “Maintaining close relationships has been difficult and frustrating for me. |  |  |  |  |
| 53 | “I live life one day at a time and don't really think about the future. |  |  |  |  |
| 54 | “In general, I feel I am in charge of the situation in which I live. |  |  |  |  |
| 55 | “I am good at managing the responsibilities of daily life. |  |  |  |  |
| 56 | “I sometimes feel as if I've done all there is to do in life. |  |  |  |  |
| 57 | “For me, life has been a continuous process of learning, changing, and growth. |  |  |  |  |
| 58 | “I think it is important to have new experiences that challenge how I think about myself and the world.” |  |  |  |  |
| 59 | “People would describe me as a giving person, willing to share my time with others. |  |  |  |  |
| 60 | “I gave up trying to make big improvements or changes in my life a long time ago. |  |  |  |  |
| 61 | “I tend to be influenced by people with strong opinions. |  |  |  |  |
| 62 | “I have not experienced many warm and trusting relationships with others. |  |  |  |  |
| 63 | “I have confidence in my own opinions, even if they are different from the way most  other people think.” |  |  |  |  |
| 64 | “I judge myself by what I think is important, not by the values of what others think is  important.” |  |  |  |  |
| 64b | Some people wander through life, but I am not one of them. |  |  |  |  |

**SECTION E: PERSONAL & FAMILY FACTORS THAT MAY CONTRIBUTE TO RESPONDENT’S BEHAVIOUR**

**1. FAMILY APGAR (FAMILY FUNCTIONING) QUESTIONS**

| **S/N** | **Items** | **Response (please choose/tick only one)** | | |
| --- | --- | --- | --- | --- |
|  |  | **Almost always (2)** | **Sometimes (1)** | **Hardly ever (0)** |
| 65 | I am satisfied with the help that I receive from my family when something is troubling me. |  |  |  |
| 66 | I am satisfied with the way my family discusses items of common interest and shares problem-solving with me. |  |  |  |
| 67 | I find that my family accepts my wishes to take on new activities or make changes in my lifestyle. |  |  |  |
| 68 | I am satisfied with the way my family expresses affection and responds to my feelings, such as anger, sorrow, and love. |  |  |  |
| 69 | I am satisfied with the amount of time my family and I spend together. |  |  |  |

70. How will you best describe the relationship between your parents, i.e., as husband and wife? 1. Cordial 2. They manage to get along 3. Indifferent (they don’t just care) 4. Terrible/bad (always fighting) 5. Not Applicable (NA)

71. How will you best describe the relationship between your Mum and you- 1. Cordial/ Get along very well 2. We managed to get along 3. Indifferent 4. Terrible/bad 5. Not Applicable (N/A)

72. How will you best describe the relationship between your dad and you- 1. Cordial/ Get along very well 2. We managed to get along 3. Indifferent 4. Terrible/bad 5. Not Applicable (N/A)

73. If you found yourself in emotional trouble, who will you readily talk to (1) Father, (2) Mother, **(**3) Teacher, (4) Aunt/Uncle, (5) Religious mentor, (6) Friend/ Mate, (7) Other- ……….

74. Does your Father TRY to create time to be with you and listen to you, etc.? (1) No (2) Yes (3) NA

75. Does your father give you moral/religious instruction? (1) No (2) Yes (3) NA

76. Does your Mother TRY to create time to be with you and listen to you, etc.? (1) No (2) Yes (3) NA

77. Does your mother give you moral/religious instruction? (1) No (2) Yes (3) NA

78. Would you consider yourself a deeply religious person- (1) No (2) Yes (3) Not sure

79. Please rate your involvement in spiritual activities (praying, reading the Word, fellowshipping, etc.)

1. Much involved 2. Moderately involved 3. Minimally/ rarely 4. Not involved

80. Please choose ONE option that best describes the social group you belong to**:**

a. I have no real close friends (1) b. I have a small group of close friends (2). I have a large group of friends with a few close friends (3). I have a large group of friends with no real close friends (4)

81. Which of these do you sometimes take/do- a. ALCOHOL- (1) No (2) Yes

b. SMOKING- (1) No (2) Yes c. HARD DRUGS/ SUBSTANCES- e.g. ……. (1) No (2) Yes
